# Simulation approach for common female cancers: a brief review

**DOI:** 10.3389/fonc.2025.1479225

**Published:** 2025-07-09

**Authors:** Ying Shen, Chan Li, Mao-Yan Tang, Zhen-Yu Huang

**Affiliations:** ^1^ Department of Obstetrics and Gynecology, Heilongjiang University of Chinese Medicine, Harbin, China; ^2^ Department of Obstetrics and Gynecology, The First Affiliated Hospital of Heilongjiang University of Chinese Medicine, Harbin, China; ^3^ School of Humanities and Social Sciences, Harbin Engineering University, Harbin, China

**Keywords:** simulation approach, common female cancers, Monte Carlo method, agent-based modeling, computational biology

## Abstract

Simulation approach involves the use of computers and mathematical models to simulate real systems for experimentation or tests that evaluate the behavior and performance of a system or predict the results of various hypothetical scenarios. Due to its rapid development in the context of cancer, we introduce commonly used cancer simulation approach, and review the application of these approach in common cancers of women, such as breast, cervical, ovarian and endometrial cancers, to provide new ideas and directions for cancer study as well as clinical treatment.

## 1 Introduction

Cancer, characterized by the abnormal proliferation of cells in an uncontrolled manner, is a leading cause of death worldwide. According to the WHO, approximately 19.3 million cancer cases were diagnosed in 2020, accounting for one in six deaths, and the number of cases is expected to rise to 28.4 million by 2040 (1).

The traditional methods of cancer research are mainly clinical trials, *in vitro* experiments and *in vivo* experiments, which are important but also have their own shortcomings. For example, clinical trials are not only a lengthy process, but also involve the influence of trial design, patient selection and follow-up, and complex data analysis (2). *In vitro* experiments are studied by extracting cells from patients, but cannot fully simulate the physiological environment *in vivo*, etc. (3); the main advantage of *in vivo* experiments is the use of replacement animals, but they may be affected by individual differences, environmental factors, time, and ethical issues, etc. (4).

Compared to traditional research methods, cancer simulation is a valuable research direction that can provide an ethical, rapid, and cost-effective approach to various hypotheses and predictions for cancer prevention, diagnosis, and treatment (5). Simulation modeling involves the use of computer and mathematical models to simulate real systems for experiments or tests that evaluate the behavior and performance of a system or predict the outcomes of various hypothetical scenarios (6).

Cancer simulation is capable of investigating the complex interactions of multiple factors such as molecular pathways, cell proliferation, and tissue microenvironment (2), assessing patient clinical data and developing individualized screening strategies through a multiscale approach (7), obtaining accurate, rigorous, and reproducible predictions of spatiotemporal progression of cancers (8), and mimicking the growth dynamics of cancer genesis, metastasis, tumor angiogenesis or immune microenvironment formation processes (9).

Therefore, this paper provides new ideas for future cancer research and clinical treatment by summarizing existing simulation approaches and presenting the results achieved in current common cancer types in women, respectively, as well as perspectives for the future. The rest of the paper is organized as follows. Section 2 describes the methods. Section 3 shows the results of breast, cervical, ovarian, and endometrial cancer simulations. Section 4 is our discussion. Section 5 is the conclusion.

## 2 Main methods of cancer simulation modeling

With the advent of multidisciplinary collaborations, cancer simulation modeling has become a powerful tool for in-depth mechanism research, the development of improved therapeutic strategies, and the prediction of cancer outcomes. In the existing literature, Monte Carlo simulation, multi-scale modeling and simulation, agent-based modeling, and other computational biology methods are currently in common use for cancer simulation modeling.

### 2.1 Simulation modeling based on Monte Carlo method

The Monte Carlo method enables systems to be modeled according to first principles and is a form of computation that uses random sampling and iteration to model the evolution of a physical or biological system, involving the use of a probability distribution function to make decisions. Geant4 is the commonly used Monte Carlo platform, along with joint modeling with other platforms or computer algorithms and codes. For example, Masurel et al. applied a kinetic Monte Carlo algorithm to directly simulate the kinetic equations with a DSMC approach to develop a theoretical model of the controlled thermostatic dynamics of tumor growth, providing some clues as to how tumors respond to mimic the cyclic dual disruption of vaccinations and chemotherapy (10). Chatzipapas et al. used the Geant4-DNA Monte Carlo toolkit to simulate the computational environment of human cancer cell radiation to gain a more conclusive understanding of radiation-induced biological damage (11). Based on Indian buffet process (IBP) modeling, Ogundijo demonstrated that the use of a Monte Carlo modeling algorithm SeqClone for the de-convolution of variant allele fractions (VAFs) from tumor sequencing data was an effective solution to address the genetic heterogeneity of tumor samples (12).Liu R et al. designed and implemented a coupled simulation method by merging the cell biology simulation platform-CC3D and the open-source Monte Carlo platform-Geant4 to develop a “bridging” module RADCELL, which combines radiative transport models and cell biology models, allowing us to simulate the dynamics of biological tissues in the presence of ionizing radiation. This provides a framework to quantify the biological consequences of radiation therapy, and it can be applied to study the use of radiation therapy in vascularized tumors (13). **Table 1** shows more biotechnological cases of how to use Monte Carlo Simulation for radiation therapy optimization, tumor growth modeling, and genetic heterogeneity analysis.

**Table 1:** Biotechnological Cases of Monte Carlo Simulation.

Application	Biotechnological Cases	Reference
Radiation Therapy Optimization	Case: Head and Neck Cancer Survival Prediction Study. Method: Monte Carlo method applied via Bootstrap resampling (1000 times) for model validation. This method randomly generates splits for training and validation sets to estimate the confidence interval of Harrell’s C-index. Parameters (1): CT converted to Hounsfield Units (HU), PET converted to Standardized Uptake Value (SUV); images resampled to 1mm isotropic voxels (2); Extraction of 42 features (21 CT + 21 PET) (3);Bags of Bags of Visual Words (BoVW) clustering using Gaussian Mixture Models.	(14)
	Case: Cross-dose assessment for intrahepatic tumors in Radiopharmaceutical Therapy (RPT). Method: Monte Carlo method implemented via Geant4 hybrid geometry simulation for cross-dose evaluation. Parameters (1): ICRP110 adult male voxel phantom (2.14mm³ voxels), with cuboidal tumors implanted in liver (1×1×1 to 3×3×3 voxels) (2); Adenocarcinoma (9.9% H, 26.9% C, 56.9% O) (3). ^90^Y (pure β emitter, max tissue range 11mm), ^177^Lu (β emitter + 113keV/208keV γ rays, β max range 2.2mm) (4); Specific Absorbed Fraction (SAF), S-value (mGy/MBq/h); validated against ICRP133, OpenDose & IDAC-Dose2.1	(15)
Tumor Growth Modeling	Case: Formation of tumor spheroids by GFP-HEK-293 cells in 3D-printed PDMS microwells. Method: Monte Carlo simulation used to predict tumor spheroid formation process within microwells. A 30×30×30 grid model established in MATLAB simulates cell diffusion, division & interaction, gravity, and directionality. Parameters (1): Microwell diameter: 400/600/800 μm (2); Cell seeding density: 500/1000/1500 cells/well (3); Centrifugation conditions: 1000g, 5 minutes (to distribute cells) (4); Surfactant: Anti-adhesion solution pretreatment (5); Culture time: 7 days (6); Spheroid size: 454 ± 15 μm (400 μm well), 459 ± 7 μm (600μm well), 451 ± 18 μm (800μm well) (7); Sphericity: >0.8; Cell viability: >90%.	(16)
	Case: Mouse brain glioma model Method: Multiscale Monte Carlo simulation (TOPAS + CompuCell3D) investigating the influence of tumor growth and vascular spatial distribution on Microbeam Radiation Therapy (MRT) efficacy. Parameters (1): Tumor volume: Day 12 stage 0.019mm³ → Day 20 stage 0.195mm³ (2); Vascular spatial homogeneity decrease rate: Day 12 stage 6.2% → Day 20 stage 18.5% (3); pO_2_ diffusion coefficient: 2×10³ μm²/s (4); Tumor cell oxygen consumption rate: 10 × normal cells (0.6 mmHg/s) (5); Irradiation depth: 5.5mm.	(17)
Genetic Heterogeneity Analysis	Case: Sequential Monte Carlo (SMC) algorithm for inferring tumor subclone genotype and proportion matrices. Method: Utilizes a state-space model and Indian Buffet Process (IBP) to process variant allele fraction (VAF) data, addressing tumor heterogeneity. The algorithm supports dynamic addition of new SNV data to optimize estimation, suitable for large-scale genomic locus analysis. Parameters (1): Simulated dataset (sequencing depth r ∈ {50, 200, 1000} (2); Number of subclones C ∈ {3, 4, 5} (3); Number of samples S ∈ {3, 4,…, 10}; Number of loci T ∈ {20, 40, 60, 80, 100, 5000} (4); Real chronic lymphocytic leukemia (CLL), samples: patients CLL077, CLL006, CLL003.	(12)
	Case: Tumor and its microenvironment (TME), exhibiting density gradients from center, periphery, to intermediate zones. Method: Monte Carlo simulations analyze the impact of tumor hypoxia heterogeneity on radiotherapy. Parameters (1): Using a 200×200 square lattice divided into multiple shells as an example, simulating scenarios where cell removal probability differs per shell (2); Parameter r is defined as the rate of decrease in removal probability from one shell to the next (towards the center) (3); When r=0.1 and divided into five shells, removal probabilities are p=[0.66, 0.73, 0.81, 0.9, 1] (4); Simulations calculate the lattice percolation threshold pc for different r values, finding pc increases linearly with r (e.g., pc≈0.5915 at r=0, pc=0.608 at r=0.1, pc=0.642 at r=0.2), indicating tumor hypoxia heterogeneity affects radiotherapy efficacy.	(18)

### 2.2 Multi-scale simulation modeling

**Figure 1:**
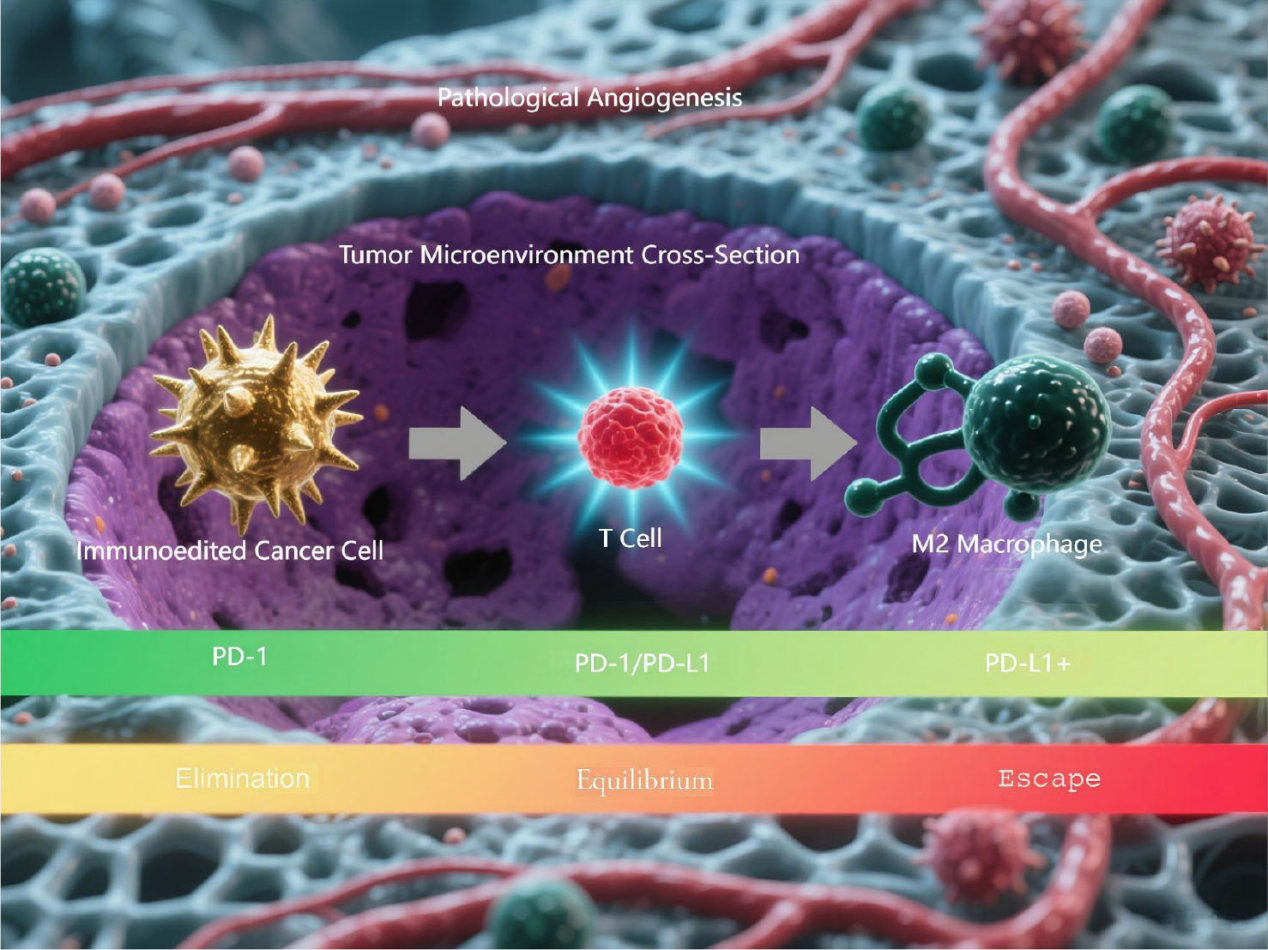
Three-phase cancer immunoediting process. This is a computational model simulating the three-phase cancer immunoediting process. The illustration demonstrates the 3D spatial architecture of the tumor microenvironment cross-section and the dynamic progression through the elimination, equilibrium, and escape phases.

Tumor growth encompasses multi-cellular dynamics at different spatial and temporal scales for intracellular and extracellular processes, and the existing literature mainly applies the Statecharts language, SimuLife visualization and other methods, and simulation tools such as Rhapsody, Matlab, IncuCyte and CompuCell3D simulation are used to investigate the multi-scale simulation study of tumor cell dynamics. For example, Bloch used the Statecharts language and Rhapsody tool to create comprehensive 3D models of solid tumors and their microenvironments, and combined with SimuLife visualization to simulate the angiogenesis process in tumors and their microenvironments (19). Bouchnita et al. modelled the physiological process of tumor growth at different scales to study the effect of acquired mutations in the EGFR/ERK pathway on single-cell dynamics (20). Lima et al. developed a coarse-grained two-scale ABM via Matlab and IncuCyte software to simulate tumor cell motility, growth, and phenotypic transformations, and thus to study the interactions between tumors and glucose consumption (21). Jafari et al. developed a multiscale model of 2D tumor vascular growth to couple multiple time and length scales through the CompuCell3D simulation environment to explore the consequences of targeted receptor inhibition in tumor development (22) (**Figure 1**). The essence of multi-scale integration lies in identifying “scale-bridging molecules”. For instance, in reference (20), the scale-bridging molecules enabling multi-scale integration are the epidermal growth factor receptor (EGFR), its ligand (EGF), and downstream signaling molecules (e.g., MEK and ERK), which play central roles in extracellular and intracellular signal transduction processes; while in reference (21), the scale-bridging molecule facilitating multi-scale integration is glucose, which, as a key nutrient for cell growth and metabolism, bridges the tissue scale and the cellular scale.

### 2.3 Agent-based modeling

**Figure 2:**
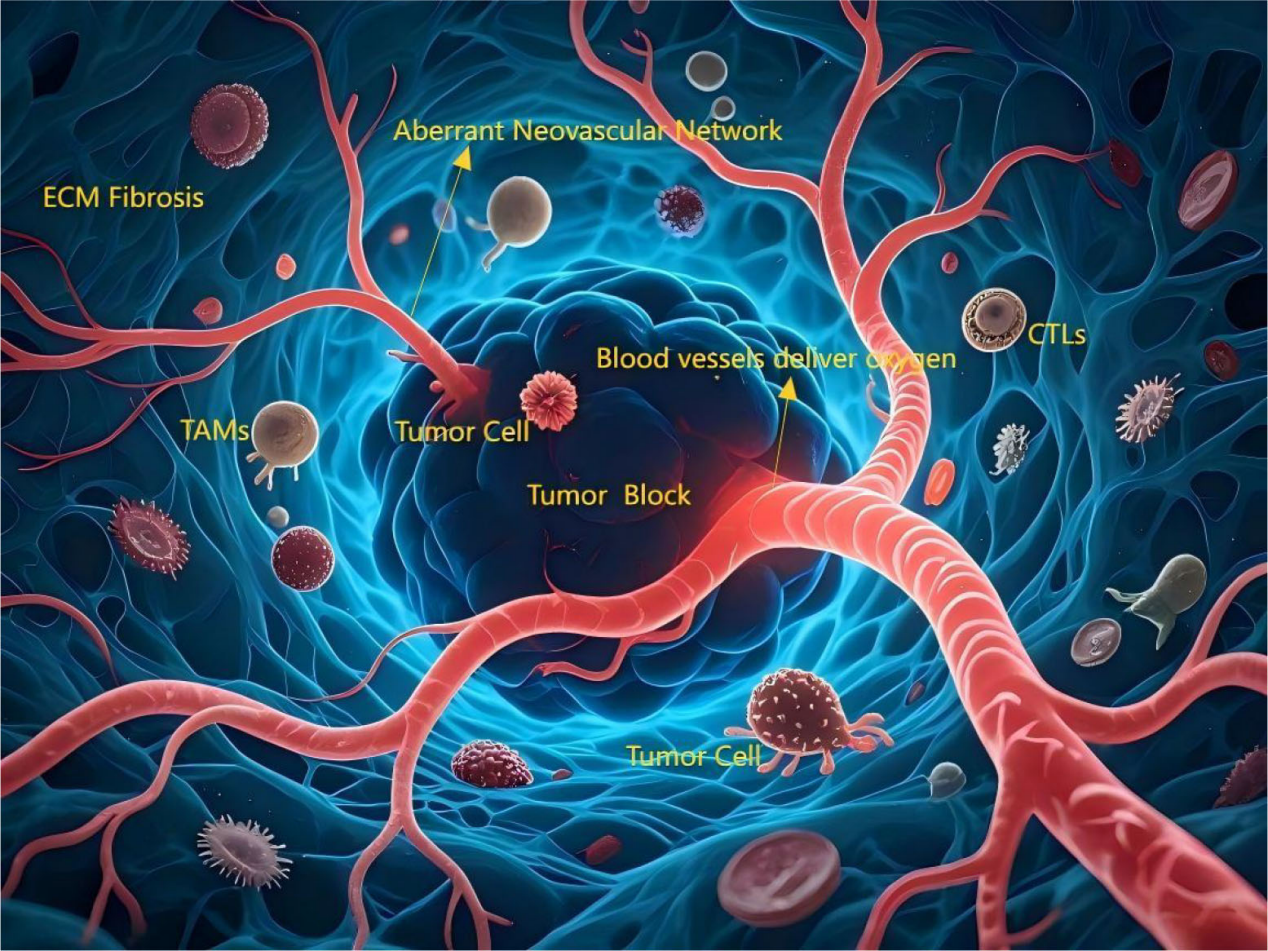
The angiogenesis process in tumors and their microenvironments. The tumor core region (dark, irregular cellular mass) is surrounded by an aberrant neovascular network (tortuous, leaky, immature). Blood vessels deliver oxygen along a gradient (red [high] → deep blue [low]), with the core exhibiting significant hypoxia (deep blue). Actively proliferating tumor cells (brightly stained, mitotic figures) localize near vasculature, while invasive tumor cells (amoeboid morphology, pseudopods) migrate toward hypoxic zones and tissue boundaries. Diverse immune cells infiltrate: Tumor-Associated Macrophages (TAMs, large volume, peritumoral), Cytotoxic T Lymphocytes (CTLs, attempting tumor cell contact but partially inhibited). The Extracellular Matrix (ECM, reticular fiber structure) appears thickened/disorganized (fibrotic).

Agent-based modeling (ABM) is a computational model used to simulate the actions and interactions of autonomously conscious agents, which is capable of reproducing and predicting complex phenomena (23). ABM-based cancer simulation can describe biological phenomena in an intuitive and modular multi-scale manner (24, 25) and study aspects of angiogenesis (26, 27) and immune response (28, 29), while Netlogo, Python, etc., are the main software used to carry out cancer simulation studies using ABM. For example, Ponce-de-Leon et al. proposed PhysiBoSS 2.0 based on the stochastic Boolean and ABM modeling frameworks for studying interactions between the microenvironment, signaling pathways controlling cellular processes and population dynamics, as well as drug action and synergism in cancer cell line models, and introduced the Python package for handling and processing simulation outputs (30).Jalalimanesh et al. used ABM as a new approach to calculate the optimal dose of radiation therapy for tumor, modeling the process of tumor angiogenesis and oxygen diffusion to simulate the effect of radiation therapy on tumor angiogenesis (31).Rojas-Dominguez et al. used Netlogo to build a cancer immunoediting model through logical functions to simulate the confrontation between cancer and immune response, in order to understand the interaction between the immune system and tumor cells occurring in the tumor microenvironment (32).Rivera et al. simulated peritoneal implantation, intravascular and hematogenous metastasis of ovarian cancer to distant organs in the OCMetSim-Single Cells and OCMetSim-Spheroids models by Netlogo, which was used to investigate the effect of RAC1 gene expression on metastasis of ovarian cancer (33) **Figure 2**.

## 3 Simulation modeling applications for malignant tumors in women

**Figure 3:**
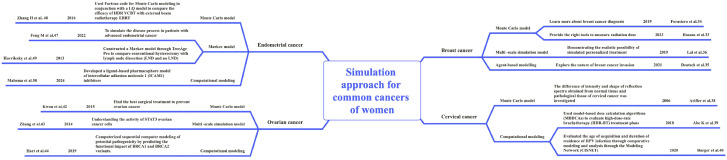
Simulation modeling applications for different cancer types.

In this section, we mainly introduce the research methods and results of simulation models in breast cancer, cervical cancer, ovarian cancer and endometrial cancer in recent years (see **Figure 3**).

### 3.1 Breast cancer

**Figure 4:**
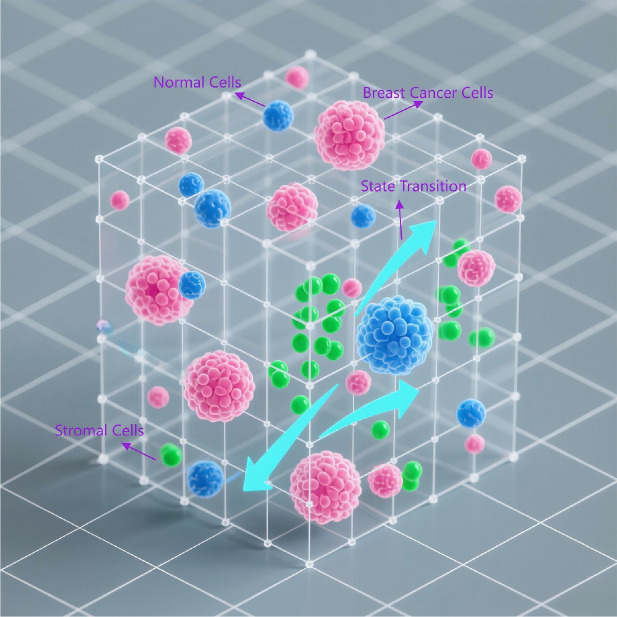
Bio-Lattice Gas Cellular Automaton (BIO-LGCA) model. This is a Bio-Lattice Gas Cellular Automaton (BIO-LGCA) model based on Langevin dynamics, modularly integrated to simulate invasive plasticity in breast cancer. Within the 3D lattice space: (1)Pink nodes represent breast cancer cells; (2) Blue nodes denote normal cells; (3) Green nodes indicate stromal cells; (4) Probabilistic transition arrows between lattice points model cell state transitions.

Breast cancer is one of the most common malignant cancers in women worldwide, with 80–90% of patients surviving more than 5 years after diagnosis (34). It has been reported that the incidence of breast cancer in China is on the rise (35), but the 5-year survival rate is only between 40 and 60 percent (36). In the simulation study of breast cancer, simulation modeling can not only simulate complex multicellular phenomena, but also optimize clinical treatment, making individualized treatment possible. For example, Hassan et al. simulated the luminous flux and diffuse reflectance distributions in normal and cancerous breast tissues exposed to planar and Gaussian NIR beam shapes by MCML and MCXLAB calculations based on the GPU-based Monte Carlo eXtreme model, which provides the knowledge needed to improve the quality of dosimetry data and can help clinicians choose the best tool for measuring radiation dose (37). Foraster et al. developed a Monte Carlo tool to simulate breast cancer screening procedures, which combined with the results of breast screening programs (BSPs), found that there was an overdiagnosis of between 7 and 20 percent and that it was associated with ductal carcinoma *in situ* (38).In addition, Deutsch et al. constructed a biological lattice-gas cell automaton (BIO-LGCA) based on Langevin models (see **Figure 4** as an example), using a code of equations that are combined in a modular fashion to simulate complex multicellular phenomena, elucidating the nature of the recently discovered invasive plasticity of breast cancer cells in a heterogeneous environment (39).To further explore breast cancer-specific mechanisms and therapeutic approaches, Lai et al. for the first time combined relevant bispecific mechanisms and multi-type individual patient data with pharmacokinetics and multi-scale dynamics in a mechanical and multi-scale manner to conduct a personalized computer simulation of breast cancer therapy, demonstrating the realistic possibility of simulating personalized therapy (40).

### 3.2 Cervical cancer

Cervical cancer is a common gynecological malignancy, with cervical cancer *in situ* occurs at the age of 30~35 years, and that of invasive carcinoma occurs at the age of 45~55 years. Due to the near-universal application of cervical cytology screening in recent decades, cervical cancer and pre-cancerous lesions can be detected and treated at an early stage, and its morbidity and mortality rates have been significantly reduced (41). In cervical cancer, simulation modeling has demonstrated the feasibility of early cervical cancer screening on a therapeutic basis, making the realization of early diagnosis and optimal treatment called possible. Using Monte Carlo modeling, Arifler et al. simulated a range of changes in the optical properties of normal and highly dysplastic cervical tissues by spectral measurements and provided a quantitative understanding of the specific contribution of different epithelial and stromal optical parameters to the overall spectral response, successfully describing the differences in the intensity and shape of reflectance spectra obtained from normal and CIN3+ tissue sites (42).Abe et al. used model-based dose calculation algorithms (MBDCAs) to evaluate high-dose-rate brachytherapy (HDR-BT) treatment regimens in seven patients with cervical cancer (43). Burger et al. evaluated the age of acquisition and duration of residence of HPV infection through comparative modeling and analysis through the Modeling Network (CISNET), which showed that 50% of unscreened women were infected with HPV between the ages of 19 and 23 years and that the time from HPV acquisition to cervical cancer was 17.5 to 26 years. It also elucidated the important factors of vaccination and cervical cancer screening and emphasized the value of comparative models in evaluating public health policy (44).

### 3.3 Ovarian cancer

Ovarian cancer is a malignant cancer with the highest mortality in women. Due to its insidious onset, approximately 70% of patients are already in an advanced stage when they are diagnosed (45). Multi-scale models combining mathematical methods, clinical data and computer code modeling are widely used in ovarian cancer. Kwon et al. simulated opportunistic salpingectomy as a preventive strategy for ovarian cancer based on the Monte Carlo model and found that salpingectomy reduced the risk of ovarian cancer by 39.8% compared to hysterectomy alone. Compared with tubal ligation, the risk was 29.2% lower (46). Zhang et al. simulated IL-6-stimulated ovarian cancer cell proliferation, migration, and apoptosis, as well as STAT3 pathway activation processes, based on a multi-scale ovarian cancer model, and the simulation results were consistent with recent experimental evidence that STAT3 ovarian cancer cells have high levels of survival and drug resistance (47). Hart et al. proposed a variant-based functional assessment and the computerized sequential computer modeling of potential pathogenicity by predicting the functional impact of BRCA1 and BRCA2 variants. The author showed that the functional studies of variants of BRCA1 coincided with those of BRCA2, and that the 130 destructive and potentially pathogenic variants identified may significantly increase the risk of developing breast, ovarian, and other cancers (48).

### 3.4 Endometrial cancer

Endometrial cancer is a prevalent gynecologic malignancy in which women have a risk of developing endometrial cancer of approximately 3% (49), and with an average total cost per patient per month of $17,210 prior to treatment and an average of $6,859 during treatment (50), the financial burden is an urgent issue that needs to be addressed. Therefore, in endometrial cancer, simulation modeling focuses on treatment and finding the best cost treatment options. One piece of evidence suggests that Feng et al. used TreeAge Pro 2020 software to develop a Markov model to simulate the disease process in patients with advanced endometrial cancer, and found that in pretreated patients with advanced endometrial cancer, lenvatinib plus pembrolizumab was not cost-effective compared with chemotherapy (51).Zhang et al. used Fortran code for Monte Carlo modeling in conjunction with a linear-quadratic (LQ) model to compare the efficacy of high-dose-rate (HDR) vaginal cuff brachytherapy (VCBT) with external beam radiotherapy EBRT, and found that for homogeneous distribution of cancer cells and radiation-resistant normal tissues, radiobiological outcomes of HDR VCBT did not show superiority over EBRT (52).Havrilesky et al. constructed a Markov model through TreeAge Pro to compare conventional hysterectomy with lymph node dissection (LND and no LND), and used Monte Carlo to explain the uncertainty of the model, showing that LND was much less likely to be cost-effective for patients with grade 2 and 3 endometrial cancer (53).Mahema et al. developed a ligand-based pharmacophore model of intercellular adhesion molecule 1 (ICAM1) inhibitors by kinetic simulation and quantum mechanics, continuously screened a variety of anticancer drugs exhibiting pharmacophore profiles that inhibit ICAM1, and combined free-energy and kinetic simulations to show that lanreotide-ICAM1 complexes, when used in the treatment of endometriosis, may delay or prevent endometrial cancer (54).

## 4 Discussion

### 4.1 Simulation approach is an important development for women’s cancers

Monte Carlo methods, multi-scale simulation modeling and ABM are commonly used simulation methods for the study of common cancers in women, and the main simulation tools include Matlab, Rhapsody, IncuCyte, Netlogo, Python, and Geant4, etc. From the received literature, it can be seen that women’s cancer simulation has evolved from simulation of tumor growth models to more detailed simulation of how blood vessel growth, oxygen distribution, immunity and the microenvironment affect tumor cell proliferation and invasion, including solving the problem of the economic burden of cancer treatment, providing computational support for women’s cancer clinical treatment, cancer screening, optimal radiation dosage, and reduction of treatment costs. In the information age, women’s cancer simulation has become an important development in computational medicine, and even computational biology, providing important new directions for women’s cancer treatment and accelerating the speed at which individualized treatment becomes possible.

### 4.2 Trends in women’s cancer simulation research

The integration of simulation research methods with traditional or emerging research methods is an important trend in women’s cancer simulation research (1). Integration of simulation modeling and clinical trials, Chen et al. developed a model of drug-directed therapy for pancreatic cancer and explored the impact of immunotherapy on patient survival based on simulation studies using Monte Carlo methods and validation of the model using clinical data from two patients (55) (2); Integration of simulation modeling and biological experiments, Hashemi et al. studied the effect of gold nanoparticles (GNPs) combined with electron brachytherapy in an ocular tumor model through Monte Carlo modeling. It was verified through experiments, showing that the concentration of GNPs could increase the target dose and could be used as a dose enhancer in the tumor area. This is expected to be a beneficial method for the treatment of superficial ocular lesions and tumors (56) (3); Integration of simulation modeling and machine learning methods, Lu et al. developed a deep-learning-based algorithm for tumor origin assessment, which trained a model with the whole-section images of known primary tumors to simultaneously identify whether a tumor was primary or metastatic and predict its site of origin (57).

## 5 Conclusions

We introduce commonly used cancer simulation research methods and tools, and review the application of these methods and tools in common women cancers such as breast, cervical, ovarian and endometrial cancer. At the same time, we found that the study of women cancer simulation is helpful to reduce research costs, help to understand the micro-mechanisms of processes such as cancer cell proliferation, invasion and metastasis, angiogenesis, and the micro-environment, as well as help to identify optimal therapeutic strategies and explore personalized treatments, which constitutes an important research content and research direction of computational medicine. Finally, in the discussion, we further proposed that the integration of simulation research with other types of research methods as a future trend for simulation research in women’s cancers.

With the advent of the artificial intelligence era, women’s cancer simulation research will be able to bring more important help to women’s life and health. However, unfortunately, this study mainly focuses on the importance and development trend of simulation methods in women’s cancer research, without a detailed description of the specific application of specific simulation tools and the simulation process. In future studies, we will try to use specific women cancers (e. g. ovarian cancer) as case studies, and use a combination of simulation methods, experimental studies and clinical observations to carry out the studies, as well as to simulate and compare the efficacy of various drugs and establish prognostic models. In the process, we will try to provide a comprehensive and specific cancer simulation model as well as an introduction to the operation process, with a view to providing researchers and clinicians with research assistance.
